# Differential trends and patterns of sociodemographic disparities in burden of mental disorders, substance use disorder and self-harm across age groups: ecological study in 204 countries using the Global Burden of Disease Study 2019

**DOI:** 10.1192/bjo.2024.26

**Published:** 2024-04-19

**Authors:** Minjae Choi, Joshua Kirabo Sempungu, Eun Hae Lee, Yo Han Lee

**Affiliations:** Institute for Future Public Health, Graduate School of Public Health, Korea University, Seoul, Republic of Korea; and Department of Preventive Medicine, Korea University College of Medicine, Seoul, Republic of Korea; Department of Preventive Medicine, Korea University College of Medicine, Seoul, Republic of Korea; and Program in Public Health, Graduate School, Korea University, Seoul, Republic of Korea; Department of Preventive Medicine, Korea University College of Medicine, Seoul, Republic of Korea

**Keywords:** Mental disorder, substance use disorder, self-harm, sociodemographic development, age difference

## Abstract

**Background:**

It is well-known that socioeconomic status is associated with mental illness at both the individual and population levels, but there is a less clear understanding of whether socioeconomic development is related to poor mental health at the country level.

**Aims:**

We aimed to investigate sociodemographic disparities in burden of mental disorders, substance use disorders and self-harm by age group.

**Method:**

Estimates of age-specific disability-adjusted life years (DALY) rates for mental disorders, substance use disorders and self-harm from 1990 to 2019 for 204 countries were obtained. The sociodemographic index (SDI) was used to assess sociodemographic development. Associations between burden of mental health and sociodemographic development in 1990 and 2019 were investigated, and sociodemographic inequalities in burden of mental health from 1990 to 2019 by age were estimated using the concentration index.

**Results:**

Differential trends in sociodemographic disparities in diseases across age groups were observed. For mental disorders, particularly depressive disorder and substance use disorders, DALY rates in high SDI countries were higher and increased more than those in countries with other SDI levels among individuals aged 10–24 and 25–49 years. By contrast, DALY rates for those over 50 years were lower in high SDI countries than in countries with other SDI levels between 1990 and 2019. A higher DALY rate among younger individuals accompanied a higher SDI at the country level. However, increased sociodemographic development was associated with decreased disease burden for adults aged ≥70 years.

**Conclusions:**

Strategies for improving mental health and strengthening mental health system should consider a broader sociocultural context.

Mental illness, including mental disorders, substance use disorders and self-harm, is increasingly recognised as an important public health issue owing to its growing contribution to elevated disease burden.^1^ The life expectancy of individuals with mental illness is 7–10 years shorter than that of people without mental illness,^2^ because mental illness has an early age at onset and can also lead to significant adverse socioeconomic burdens over long-term periods.^3,5^ In 2019, the global age-standardised disability-adjusted life years (DALY) rates for mental disorders, substance use disorders and self-harm were 1566.4, 432.5 and 424.7 per 100 000 population, respectively.^6^ Total DALYs attributed to mental disorders, substance use disorders and self-harm increased from 5.4% in 1990 to 7.7% in 2019,^6^ and regional differences in changes of burden of mental disorders, substance use disorders and self-harm were also observed.^7^ As individual characteristics may not fully explain these regional differences in poor mental health,^8,9^ aspects of sociodemographic development such as sociocultural factors and mental health systems at the national level could make important contributions to some of these differences.^10,11^

A number of studies investigating burden of disease at the country level have reported that a lower level of sociodemographic development was associated with an increased disease burden, particularly lower life expectancy,^12^ all-cause disease burden,^13^ communicable diseases^13^ and non-communicable diseases burden.^14,18^ However, a growing number of studies in recent years have shown opposite findings, reporting higher disease burden in high-income countries than in low- and middle-income countries, especially for mental disorders,^5,19^ substance use disorders^19,20^ and self-harm.^21^ This suggests that the burden of poor mental health is paradoxically greater in more developed countries but lower in less developed countries and territories. If the burden of poor mental health is lower in more developed countries, we could consider poor mental health to be a ‘rich-country problem’, not a priority for less developed countries.^22^ However, these contradictory findings have not been observed in cross-national data on subjective well-being,^23^ unhappiness, sadness and worry,^22^ which are highly associated with mental illness. Given that reasons for these differences have been continuously discussed, a more in-depth understanding of what these contradictory findings mean is essential to inform future public health planning for the healthcare system at global and country levels.^23^

The regional disparities in the burden of mental disorders, substance use disorder and self-harm may be explained differently for different age groups. For example, previous studies have reported a negative association between per-capita GDP and subjective well-being in adolescents but a positive association in adults.^24^ However, it is unclear whether sociodemographic development is associated with the burden of poor mental health in a different or similar manner across different age groups. Therefore, further research is needed to clarify how sociodemographic development is associated with the burden of poor mental health by age group to enhance our understanding of population-level mental health.

This study aims to fill this research gap by using data from the Global Burden of Disease 2019 study (GBD 2019) across 204 countries and territories from 1990 to 2019: (a) to describe the burden of mental disorders, substance use disorders and self-harm by a country's level of sociodemographic development; (b) to investigate differential patterns in correlations between these mental health conditions and sociodemographic development across age groups; and (c) to investigate differential trends in sociodemographic disparities in the burden of mental disorders, substance use disorder and self-harm across age groups.

## Method

### Data source and extraction

We used estimates from GBD 2019 to analyse the burden of mental disorders, substance use disorders and self-harm across age groups from 1990 to 2019. GBD 2019 provides compressive and systematic estimates of health outcomes, including incidence, prevalence, mortality and DALYs for 369 diseases and injuries, by age (23 age groups) and sex for 204 countries and territories from 1990 to 2019.^25^ DALY is the sum of years of life lost owing to premature mortality and years lived with disability. Diseases and injuries were classified into four hierarchical levels from three broadest-cause groups at level 1 (communicable, maternal, neonatal, and nutritional diseases; non-communicable diseases; and injuries) to 301 disease and injury causes at level 4^25^ to make comparisons of disease burden between locations; this was possible because the estimation methodology of GBD 2019, such as case definition, data collection and statistical methods, was coherently applied in each region.^26^ Briefly, the GBD study used multiple data, including vital statistics, disease registries and other sources, assessed their quality to minimise biases in each source, and used consistent statistical modelling methods, thereby generating estimates with 95% uncertainty intervals and providing opportunities for comparative health assessments between countries.^25^ The GBD 2019 employed standardised estimation methods, including the cause of death ensemble model, spatiotemporal Gaussian process regression and DisMod-MR.^25^ Details of the estimation methods used to derive each measure in GBD 2019 have been published elsewhere.^25^

In this study, we used estimates of DALY rates for mental disorders and substance use disorders at level 2 and for anxiety disorders, attention-deficit hyperactivity disorder, autism spectrum disorder, bipolar disorder, conduct disorder, depressive disorder, eating disorder, idiopathic developmental intellectual disability, schizophrenia and other mental disorders, alcohol use disorder, drug use disorder and self-harm at level 3 based on the ICD-9 from 1990 to 1995 and the ICD-10 from 1996 to 2019 (Supplementary Table 1 available at https://doi.org/10.1192/bjo.2024.26).

### Categorisation for age and sociodemographic index (SDI)

We used DALY rates per 100 000 population for the 15 causes stratified by age group in 204 countries and territories. Based on previous studies and a classification provided by GBD 2019, populations were categorised into the following groups: 10–24 years (youth), 25–49 years (young adults), 50–69 years (middle adults) and ≥70 years (older adults).^27^ To investigate the association between disease burden and sociodemographic development, SDI was used from 1990 to 2019. This index is a composite measure to quantify the sociodemographic development of each country and territory included in the GBD study.^12^ The SDI was calculated as the geometric mean of three indicators (lag distributed income per capita, average educational attainment for those aged 15 years and older, and total fertility rate for those under the age of 25 years). It ranges from 0 to 1, with a lower value indicating a lower level of development and *vice versa*.^12,25,28^ We divided 204 countries into the following five categories based on SDI level from GBD 2019: low SDI, low-middle SDI, middle SDI, high-middle SDI and high SDI (Supplementary Table 2). All data for this study were obtained from the Global Health Data Exchange.^6^

### Statistical analysis

First, we conducted a descriptive analysis to examine age differences in DALY rates per 100 000 population and percentage changes in mental disorders, substance use disorders, self-harm, ten specific mental disorders, and two specific substance use disorders with 95% uncertainty intervals from 1990 to 2019. Second, we further explored differential changes in DALY rates between 1990 and 2019 by SDI quintiles (low SDI, low-middle SDI, middle SDI, high-middle SDI and high SDI) in each age group. Third, we performed a linear regression analysis to test correlations between SDI and mental disorders, substance use disorders and self-harm by age group in 1990 and 2019 with a statistical significance set at *P* < 0.05, as widely used in previous studies using GBD data.^25^ Fourth, a concentration index was used to investigate socioeconomic inequalities in mental disorders, substance use disorders and self-harm by age group, a similar approach to that used in previous studies.^29,31^ The concentration index is one of the most widely used methods for measuring inequality.^32^ This index is based on a concentration curve, wherein the x-axis indicates the cumulative proportion of individuals or groups by socioeconomic level, ranging from the lowest to the highest, and the y-axis represents the cumulative proportion of disease burden for these individuals or groups.^33^ It ranges from −1 (more concentrated disease burden in locations with low SDI) to 1 (more concentrated disease burden in countries with high SDI). We used CONINDEX modules from Stata to estimate the concentration index.^34^ The concentration index was calculated by correlating DALY rates for mental disorders, substance use disorders and self-harm with the corresponding national SDI from 1990 and 2019. We also estimated regression coefficient and concentration index values for three specific mental disorders (anxiety disorder, depressive disorder and schizophrenia) and two specific substance use disorders (alcohol use disorder and drug use disorder). All analyses and visualisations were performed with Stata 17.0 (Stata, College Station, Texas, USA) and Tableau Desktop (Tableau, Seattle, Washington, USA), both on Windows.

As this study used aggregated country-level data publicly available on the website of the GHD Exchange, it did not require approval from a research ethics board or participant consent. The authors assert that all procedures contributing to this work comply with the ethical standards of the relevant national and institutional committees on human experimentation and with the Helsinki Declaration of 1975, as revised in 2000.

## Results

### DALY rates for mental disorders, substance use disorders and self-harm by age group from 1990 to 2019

Table 1 presents crude global DALY rates for mental disorders, substance use disorders and self-harm across age groups from 1990 to 2019. Global DALY rates for mental disorders in 2019 were 1512.9, 2096.0, 2060.9 and 1678.8 in age groups 10–24 years, 25–49 years, 50–69 years and ≥70 years, respectively. DALY rates for mental disorders did not change much across all age groups between 1990 and 2019 (e.g. DALY rates for those aged 10–24 years were 1506.9 in 1990 versus 1512.9 in 2019). Among the ten specific mental disorders, the most prevalent conditions in 1990 and 2019 were depressive disorders and anxiety disorders in all age groups. High DALY rates for schizophrenia were also observed in all age groups except for those aged 10–24 years. No marked changes in these mental disorders were identified. Regarding substance use disorders, overall DALY rates were slightly changed from 1990 to 2019, whereas marked changes were observed for each subcategory of substance use disorders. DALY rates of alcohol use disorder in all age groups except for those aged ≥70 years were substantially decreased, whereas DALY rates of drug use disorder in all age groups except for those ≥10–24 years were substantially increased. In particular, DALY rates for those aged 25–49 years were the highest among all age groups in 1990 and 2019, increasing from 350.7 (95% uncertainty intervals 270.6–444.5) in 1990 to 421.5 (95% uncertainty intervals: 335.4 to 520.0) in 2019. DALY rates for self-harm showed considerable reductions across all age groups. DALY rates for those aged 25–49 years were the highest among all age groups in 1990 and 2019, decreasing from 1043.9 (95% uncertainty intervals: 927.7 to 1119.4) in 1990 to 647.7 (95% uncertainty intervals: 585.7 to 711.5) in 2019.

**Table 1:** Disability-adjusted life years (DALY) rates and 95% uncertainty intervals for mental disorders, substance use disorders and self-harm between 1990 and 2019 by age group

	10–24 years	25–49 years	50–69 years	≥70 years
Mental disorders				
1990	1506.9 (1076.8–2036.4)	2127.3 (1564.9–2765.3)	2056.2 (1528.3–2708.4)	1701.6 (1262.0–2183.3)
2019	1512.9 (1078.9–2045.7)	2096.0 (1551.1–2724.1)	2060.9 (1525.4–2710.2)	1678.8 (1244.5–2161.8)
Change %	0.4 (−0.7–1.5)	−1.5 (−2.6–0.4)	0.2 (−0.5–1.0)	1.3 (−2.4–0.2)
Anxiety disorder				
1990	410.4 (270.1–591.2)	454.6 (296.9–645.2)	433.8 (295.6–609.5)	388.3 (262.9–558.8)
2019	402.7 (265.9–582.0)	459.8 (300.2–651.7)	437.2 (298.6–613.8)	374.0 (252.8–536.2)
Change %	−1.9 (−3.8–0.1)	1.1 (−1.4–3.6)	0.8 (−0.4–2.1)	−3.7 (−5.6–1.5)
ADHD				
1990	31.1 (17.5–54.4)	11.7 (6.3–20.2)	3.2 (1.6–5.7)	0.3 (0.1–0.7)
2019	27.6 (15.5–48.2)	10.4 (5.5–18.0)	3.2 (1.5–5.7)	0.3 (0.1–0.7)
Change %	−11.2 (−13.6–8.6)	−10.9 (−14–7.9)	−0.5 (−4.7–3.7)	−5.8 (−16.1–3.5)
Autism spectrum disorder				
1990	61.8 (40.3–89.8)	55.7 (36.4–80.0)	46.8 (31.1–67.7)	37.4 (25.2–53.5)
2019	61.5 (40.1–89.7)	53.7 (35.2–77.7)	45.9 (30.6–66.0)	38.7 (26–55.3)
Change %	−0.4 (−1.6–0.7)	−3.5 (−4.4–2.6)	−1.9 (−3.1–0.9)	3.4 (2.0–4.7)
Bipolar disorder				
1990	101.2 (55.8–160.3)	151.7 (92.4–237.3)	146.8 (89.3–228.6)	98.9 (60.0–149.5)
2019	107.7 (58.3–173.2)	152.9 (92.8–240.0)	139.6 (85.1–218.4)	90.6 (55.0–138.8)
Change %	6.4 (4.3–8.4)	0.8 (−1.2–2.8)	−4.9 (−6.0–3.9)	−8.4 (−10.0–6.6)
Conduct disorder				
1990	209.9 (116.0–335.4)	n/a	n/a	n/a
2019	217.8 (120.4–347.9)	n/a	n/a	n/a
Change %	3.8 (1.7–5.7)	n/a	n/a	n/a
Depressive disorder				
1990	450.6 (290.2–666.7)	823.0 (559.1–1144.8)	923.1 (637.8–1287.4)	857.9 (590.9–1162.6)
2019	452.6 (293.1–671.5)	789.1 (533.5–1105.9)	936.7 (647.2–1309.8)	856.9 (590.5–1159.2)
Change %	0.4 (−2.4–2.7)	−4.1 (−6.5–1.8)	1.5 (0.2–2.9)	−0.1 (−1.7–1.5)
Eating disorder				
1990	51.1 (30.6–79.3)	59.9 (36.5–89.5)	n/a	n/a
2019	59.8 (35.4–92.8)	65.9 (40.4–99.0)	n/a	n/a
Change %	16.9 (14.7–19.0)	10.1 (7.6–12.7)	n/a	n/a
IDID				
1990	81.8 (43.6–133.2)	59.3 (31.0–96.9)	38.6 (20.2–62.4)	20.7 (10.9–33)
2019	76.5 (40.1–125.7)	51.1 (26.6–84.3)	30.6 (15.6–50.2)	17.4 (9.2–28.0)
Change %	−6.5 (−10.2–3.7)	−13.8 (−17.7–11.0)	−20.9 (−25.8–17.8)	−16.0 (−20.7–11.9)
Schizophrenia				
1990	72.1 (47.7–104.1)	347.1 (246.9–451.6)	274.4 (199.5–350.4)	120.6 (86.9–155.7)
2019	69.5 (45.4–103.2)	346.9 (245.4–452.8)	278.1 (201.6–353.7)	121.0 (87.7–156.3)
Change %	−3.7 (−7.2–0.8)	−0.1 (−1.4–1.3)	1.3 (0.1–2.6)	0.3 (−1.5–2.3)
Other mental disorders				
1990	36.8 (19.3–60.6)	164.4 (103.9–251.5)	189.4 (121.3–282.9)	177.5 (119.0–256.3)
2019	37.3 (19.5–61.2)	166.1 (105.4–255.1)	189.5 (121.2–283.7)	179.9 (121.1–259.6)
Change %	1.3 (−0.7–3.2)	1.1 (−0.4–2.5)	0.1 (−0.8–0.9)	1.3 (0.3–2.5)
Substance use disorder				
1990	307.3 (226.6–399.5)	804.0 (633.7–1002.5)	546.7 (461.1–646.2)	253.5 (209.7–305.7)
2019	285.4 (208.2–372.5)	785.4 (627.9–966.9)	524.7 (439.2–631.2)	270.1 (220.2–331.9)
Change %	−7.1 (−11.0–2.8)	−2.3 (−5.7–1.8)	−4.0 (−7.7–0.2)	6.6 (2.7–10.1)
Alcohol use disorder				
1990	108.2 (71.4–162.2)	453.3 (352.4–586.9)	417.1 (350.2–501.2)	168.0 (134.6–211.2)
2019	83.7 (53.4–127.7)	363.9 (279.8–477.9)	346.8 (284.5–429.7)	170.8 (135.2–216.7)
Change %	−22.7 (−26.1–20.1)	−19.7 (−23.1–16.6)	−16.9 (−21.1–12.9)	1.7 (−2.7–5.3)
Drug use disorder				
1990	199.1 (146.2–264.4)	350.7 (270.6–444.5)	129.6 (104.9–160.8)	85.5 (66.8–106.5)
2019	201.7 (147.0–268.3)	421.5 (335.4–520.0)	177.9 (143.1–219.2)	99.3 (75.6–126.4)
Change %	1.3 (−4.2–7.0)	20.2 (14.3–26.8)	37.3 (30.2–44.2)	16.1 (9.6–22.4)
Self-harm				
1990	762.8 (666.9–826.7)	1043.9 (927.7–1119.4)	782.0 (706.8–842)	613.5 (565.5–661.3)
2019	454.3 (410.8–506.1)	647.7 (585.7–711.5)	460.9 (415.1–504.9)	371.2 (332.1–407.7)
Change %	−40.4 (−47–32.5)	−38.0 (−43.9–31.7)	−41.1 (−45.5–34.4)	−39.5 (−43.6–32.8)

ADHD, attention-deficit hyperactivity disorder; IDID, idiopathic developmental intellectual disability.

n/a indicates specific mental disorders for which the DALY rate was not estimated from GBD 2019; values in brackets represent the 95% uncertainty intervals.

### DALY rates for mental disorders, substance use disorders and self-harm in each SDI group by age from 1990 to 2019

**Fig. 1:**
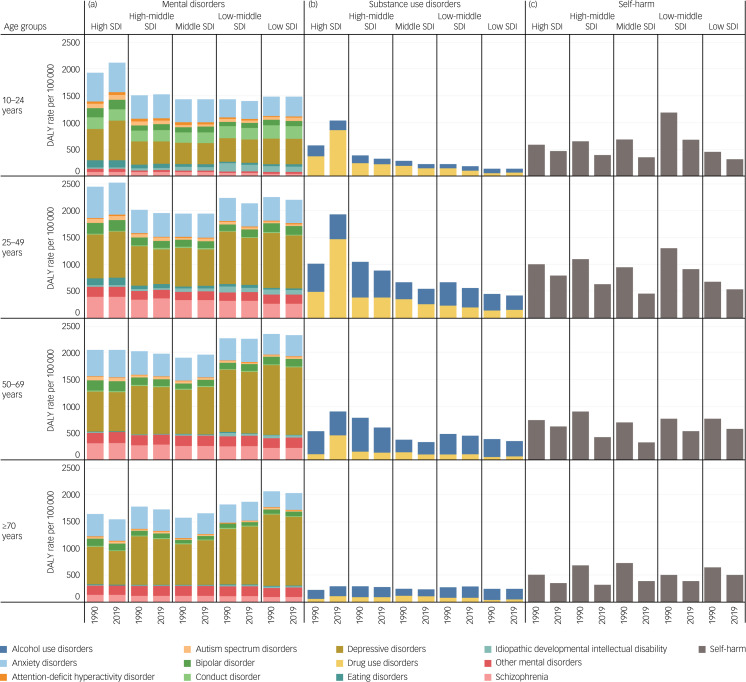
Disability-adjusted life years (DALY) rates for mental disorders, substance use disorders and self-harm between 1990 and 2019 by age group and sociodemographic index (SDI) quintiles. (a) Mental disorders. (b) Substance use disorders. (c) Self-harm.

Figure 1 and Supplementary Tables 3–6 show DALY rates for mental disorders, substance use disorders and self-harm in SDI quantiles separated by age group in 1990 and 2019. Regarding mental disorders, DALY rates in high SDI countries were higher than those in other SDI countries for those aged 10–24 years and 25–49 years, but not for those over 50 years in 1990 or 2019. Except in high SDI countries, DALY rates remained constant or decreased over the three decades in each youth and young adult group. Notably, DALY rates of depressive disorder for those aged 10–24 years in high SDI countries increased by 22.5% from 592.1 (95% uncertainty intervals: 390.7–860.6) in 1990 to 725.5 (95% uncertainty intervals: 473.6–1063.2) in 2019. DALY rates increased among high SDI countries in all age groups for substance use disorders but decreased in countries of other SDI levels from 1990 to 2019. DALY rates for substance use disorder in high SDI countries were higher than those in other SDI countries in 1990. Moreover, a considerable increasing trend for substance use disorders was observed in high SDI countries among those aged 10–24 years and 25–49 years in 2019; this could be mostly explained by increases in DALY rates for drug use disorder. Particularly, DALY rates for drug use disorder among those aged 25–49 years in high SDI countries changed by 200.3% from 493.3 (95% uncertainty intervals: 386.9–615.2) in 1990 to 1481.6 (95% uncertainty intervals: 1246.8–1738.4) in 2019, but DALY rates for drug use disorder for those aged 25–49 years in most of the other SDI levels decreased. For self-harm, DALY rates were reduced in all SDI regions during the three decades, although reductions in DALY rates in high SDI regions were smaller than those in other SDI regions for all age groups.

### Correlations of sociodemographic development with DALY rates for mental disorders, substance use disorders and self-harm in 1990 and 2019

**Fig. 2:**
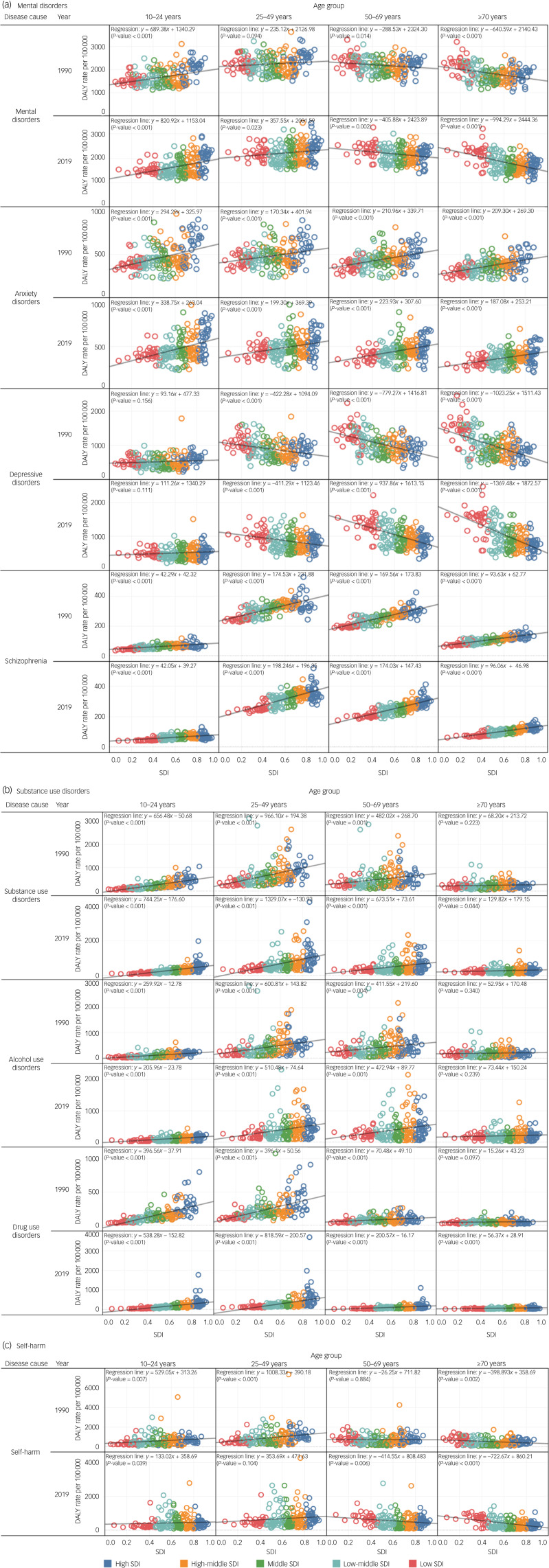
Associations between sociodemographic index (SDI) and disability-adjusted life years (DALY) rates for (a) mental disorders including anxiety disorder, depressive disorders and schizophrenia; (b) substance use disorders including alcohol use disorders and drug use disorders; and (c) self-harm between 1990 and 2019 by age group.

Figure 2 presents correlations of sociodemographic development with mental disorders, substance use disorders and self-harm. A significant association between sociodemographic development and mental disorders was identified, although different correlations were observed across age groups. A positive association between sociodemographic development and mental disorders for those aged 10–24 and 25–49 years indicated that higher sociodemographic development was associated with a higher disease burden. However, a negative association between sociodemographic development and mental disorders was observed for those aged 50–69 years and ≥70 years. Coefficients of sociodemographic development in all age groups increased from 1990 to 2019 (Fig. 2(a)). For the three subcategories of mental disorders, a positive association between depressive disorder and SDI values was observed in those aged 10–24 years. However, negative associations were observed in other age groups. Positive associations of anxiety disorder and schizophrenia with SDI values were observed in all age groups. In substance use disorders, higher sociodemographic developments were associated with higher disease burden in both 1990 and 2019. The coefficient of sociodemographic development was highest for those aged 25–49 years and second highest for those aged 10–24 years (Fig. 2(b)). There were correlations between sociodemographic development and self-harm (Fig. 2(c)). However, different correlations were observed across age groups, with a positive association in those under 50 years and a negative association in those over 60 years.

### Sociodemographic disparities in burden of mental disorders, substance use disorder and self-harm across age groups from 1990 to 2019

**Fig. 3:**
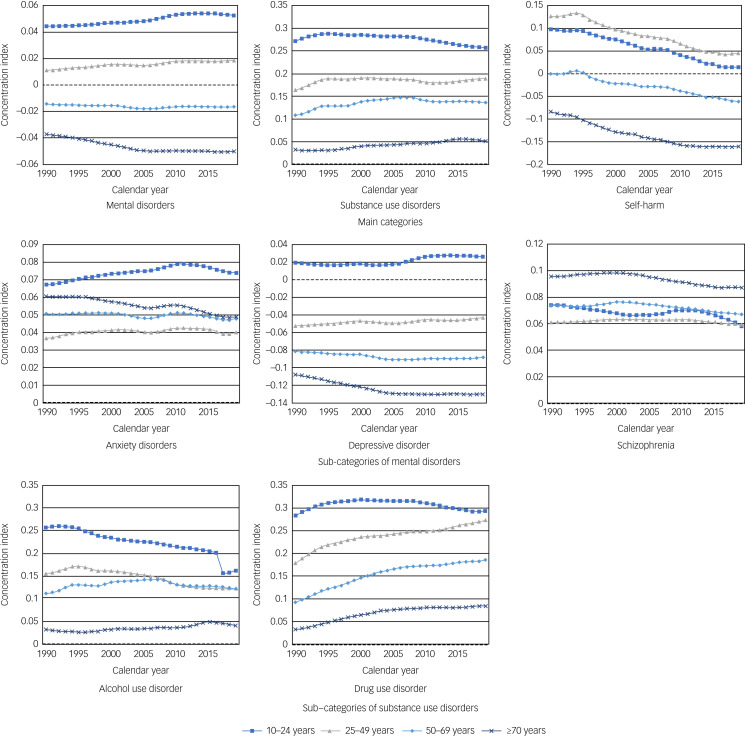
Trends in concentration index values for mental disorders, substance use disorder and self-harm across 204 countries and territories from 1990 to 2019.

Differential trends in DALY rates for mental disorders, substance use disorders and self-harm across age groups from 1990 to 2019 were found to be associated with sociodemographic disparities (Fig. 3 and Supplementary Tables 7–10). For mental disorders, a positive concentration index for ages of 10–24 years and 25–49 years and negative concentration indices for ages 50–69 years and ≥70 years were observed. This suggests that the burden of mental disorders was unequally distributed and concentrated in higher SDI regions for age groups 10–24 years and 25–49 years, whereas it was concentrated in lower SDI regions among those aged 50–69 years and ≥70 years. The ≥70 age group had a decreasing concentration index, whereas the 10–24 years and 25–49 years age groups had an increasing trend. Regarding substance use disorder, we found that the concentration index was positive in all age groups, suggesting that the burden of substance use disorder was unequally distributed and concentrated in higher SDI regions. Trends in the concentration index for substance use disorder slightly increased or remained the same from 1990 to 2019.

After stratification by subcategory of substance use disorder, however, concentration index values for drug use disorder among those aged 10–24 years, 25–49 years, 50–69 years and ≥70 years increased to 0.29, 0.27, 0.19 and 0.08 in 2019 from 0.28, 0.18, 0.09 and 0.03 in 1990, respectively. Concentration index values for self-harm exhibited markedly declining trends from 1990 to 2019 in all age groups. The concentration index based on DALY rates for the 10–24 and 25–49 years age groups decreased by near zero, suggesting that inequalities of DALY rates for self-harm associated with socioeconomic development were less concentrated in regions with higher levels of SDI from 1990 to 2019. By contrast, the concentration index based on DALY rates for the 50–69 years and ≥70 years age group were already below zero in 1990 and declined in 2019, suggesting that inequalities in DALY rates for self-harm associated with socioeconomic development were more concentrated in countries with lower levels of SDI.

## Discussion

### Main findings

Using data from GBD 2019, we investigated the association between sociodemographic development and the burden of mental disorders, substance use disorders and self-harm across four age groups (10–24, 24–49, 50–69 and ≥70 years) from 1990 to 2019. Although the burden of poor mental health remained fairly constant from 1990 to 2019, except in the case of self-harm, differential changes in the burden of poor mental health by sociodemographic development were observed across age groups. DALY rates for mental disorders, substance use disorders and self-harm were significantly correlated with sociodemographic development, although these associations were heterogeneous across age groups. Higher SDI at the country level was accompanied by higher DALY rates for poor mental health, particularly depressive disorder and substance use disorder among younger individuals. However, the opposite association was found among older age groups (e.g. ≥70 years), where increased sociodemographic development was associated with a decreased or less-increased disease burden. Furthermore, the association of sociodemographic development with the burden of poor mental health changed differentially across age groups. The burden of mental disorders and substance use disorders remained concentrated in countries with high SDI index scores, especially for youth and young adult groups.

### Interpretation of findings

For three decades, regions with high SDI have had higher DALY rates for mental and substance use disorders than low SDI regions among younger age groups, although they had lower DALY rates among older groups. In line with our findings, previous studies^19,21,35,38^ have found that the disease burden of mental disorders, substance use disorders and self-harm is paradoxically greater in less vulnerable or more developed areas. This has been referred to as the ‘vulnerability paradox’^21,35,36^ and indicates a counterintuitive association between mental health and socioeconomic resources at the country level.^21^ Specifically, our findings highlight that the vulnerability paradox is stronger among youth and young adults than older adults. People who experience mental disorders have lower subjective well-being,^22^ and previous studies have shown that children and adolescents in wealthier nations have lower subjective well-being,^24,39^ whereas the adult population has higher subjective well-being compared with their counterparts in less developed countries, consistent with our findings. Although reasons may be multifactorial and related to individual-level factors, we posit that a key explanation for differential trends and patterns of sociodemographic disparities in the burden of poor mental health across age groups is a complex interplay of two major country-level factors: differences in economic and cultural backgrounds and mental health systems.

In most high SDI countries, highly developed or productive societies may have established social values, cultural traditions, and social networks^40^ that improve their monetary value of pleasure.^20^ Generally, these societies might be characterised by higher levels of competitive individualism and indulgence, with less satisfaction obtained by pursuing basic desires. These sociocultural factors may lead to increasing pressure to achieve success and sensitivity to social failure.^21^ Accordingly, a systematic review found that economic crises such as recessions are associated with a risk of suicide, particularly in high-income countries.^41^ Moreover, we observed that DALY rates for mental disorders, particularly depressive disorder, in younger groups were increasingly concentrated in countries with high SDI levels, in contrast to trends observed in older age groups. Previous studies have found that social relationships and material circumstances, such as standard of living, are highly associated with subjective well-being in adult groups, whereas perceived life satisfaction with freedom is a factor significantly associated with subjective well-being in youth.^42^ Notably, in countries and societies whose sociocultural environments force tougher restrictions on sustained enjoyment of life than before, youth and young adults are more likely to have limited freedom and control over their lives because they are governed by social norms and their parents.^43^ In particular, educational policies intended to improve school productivity among youth or college students result in increased intensity of learning and working.^24^ Higher learning and work intensities may be linked to national socioeconomic development and educational achievement. However, these factors could also adversely affect people's mental health in various ways, such as decreased well-being or increased cognitive overload.^24,39^ Restriction of freedom and life control could be coupled to a low level of social support and integration because they are likely to be associated with autonomous activities such as using computers or mobile devices,^24^ including the rise in use of social media among young people. Previous research has suggested that internet use is more common in wealthy countries,^44^ and social media use among young people has greatly increased, reaching higher levels in this group compared with other age groups.^45^ Specifically, another study revealed that among 11 high-income countries, nearly all had experienced an increase in social media use; in some of the countries with the most significant increases, these were found to be associated with higher rates of youth suicide.^46^ Young people may be less likely to discuss emotional or personal problems or show help-seeking behaviour,^21,35^ which may lead to further deterioration of their mental health. Instead, they may turn to misuse of alcohol or drugs, which has an impact on the burden of substance use disorders. Our findings found that the increased burden of substance use disorders was highest among young people in high SDI countries. Previous studies have found that psychiatric disorders, particularly depressive disorder, coexist with substance use disorders owing to extreme alcohol or drug use and/or dependency.^47,48^ With recent advances in internet services, the availability of alcohol and drugs may have become higher owing to content and postings on social media.^49^ Given the substantial increase in use of the internet and social media, as youth and young adults are the most active users,^44,45,50^ they may be more susceptible to exposure to information on alcohol and drugs.^45^ This exposure could potentially reduce negative perceptions of the consequences of alcohol and drug use and enhance normative perceptions,^49^ which may lead to increased DALY for substance use disorders among young people, particularly in high SDI countries. Therefore, in recent years, within more competitive societies and education systems with higher material and social aspirations, these factors, including less interaction with family or friends and increased social medial use, could eventually lead to poorer mental health and explain how sociodemographic development could affect mental health among younger groups in countries with high levels of SDI. The youth mental health burden in high SDI countries is an emerging public health concern that should be addressed.

Although the DALY rates for mental health and substance use disorders were lower in lower SDI regions compared with high SDI regions, this should not lead to a belief that treatments for poor mental health conditions are priorities for only developed countries. A possible explanation is variations in mental health literacy and accessibility to professional psychiatry services among countries.^21,51,52^ These factors could influence recognition of mental distress and the probability of being diagnosed and treated for mental illness.^53^ They may also precipitate and perpetuate mental health symptoms if countries have low mental health literacy and low accessibility of professional psychiatry services.^54^ Compared with high SDI countries, where treatment uptake for poor mental health has increased since 1990,^7^ there has been a report of later onset or recognition and higher persistence of mental illness in low-income countries.^10^ Although the supportive aspects of social capital, such as social support and community engagement, could substitute for low mental health literacy and availability of mental health services in less developed countries,^21,36^ implementation of prevention and early diagnosis of mental illness is still essential for children and adolescents in low-income countries, because the youth population could buffer the long-term consequences of the mental health burden in those who need mental health treatments the most.^7^ Thus, there is a need to expand the delivery of effective prevention and treatment programmes with established efficacy to cover more of the population for the necessary duration in both high and low SDI countries.

Prevention and early intervention for mental health problems among youth and young adults are essential regardless of sociodemographic development to ensure a reduction in the mental health burden of young people and a socially and mentally healthy future adult population.

### Methodological considerations

This study provides comprehensive information on global trends and patterns in the burden of poor mental health conditions by sociodemographic development across age groups from 1990 to 2019. However, it should also be noted that interpretation of our findings needs to be cautious, considering the following methodological considerations. First, this study was based on data from GBD 2019, in which the methodology of estimating the burden of mental disorders, substance use disorders and self-harm had limitations, as generally highlighted in previous studies.^7,26^ Notably, there were variations in data quality among countries. Although estimates of GBD 2019 were calculated using a large amount of epidemiological survey data and rigorous statistical estimation methodology, estimates for some low- and middle-income countries may have been biased owing to sparsity of primary data.^7^ Cultural and religious ramifications such as social stigma may lead to underreporting of mental health problems. Although GBD 2019 used the DSM and ICD to ensure consistency in disease definitions across different epidemiological studies,^7^ these classifications might not reflect different cultural contexts in each country,^55,56^ resulting in differential misclassification. Furthermore, the estimates from GBD 2019 were based solely on disease symptoms and did not take into account psychological indicators such as undiagnosed mental health symptoms or quality of life. Therefore, the cross-cultural applicability of criteria for mental-health-related case definitions and methodology for data collection need to be considered in further GBD studies.^7^ Besides, more high-quality epidemiological research from low- and middle-income countries with more standardised methods might be needed to address these limitations. Second, this study used aggregated country-level data, which may lead to ecological fallacy and should not be interpreted as findings at the individual level. Last, we investigated the associations of a broad spectrum of burden of poor mental health and sociodemographic development with mental health, but we could not clearly explain these relationships because other contextual factors, such as cultural and health-related factors affecting mental illness, could not be used for all countries throughout the study period owing to limited data. Thus, we were very cautious when drawing conclusions based on previous studies. However, it is necessary to better understand cross-national differences in mental health burden resulting from several other factors. Further research quantifying the contribution of other contextual factors would be meaningful for developing global and country-level mental health intervention strategies to reduce the burden of poor mental health.

### Future implications

This study provides important insight into global and country-level mental health burdens across age groups by showing regional disparities in DALY rates of poor mental health, with higher SDI countries having higher DALY rates among younger groups and lower estimates among older groups than lower SDI countries. Based on our findings, three points deserve emphasis for future mental health policy and research. First, mental health strategies need to be considered within a broader sociocultural context to improve social well-being in high SDI countries. Second, in low SDI countries, mental health systems need to respond to the growing burden of poor mental health among the older population. These include improving early diagnosis and treatment for youth and reducing mental health stigma in a socially acceptable manner with further economic and social infrastructure developments. Last, more epidemiological studies should be conducted to advance our understanding of the global mental health gap and develop effective strategies in global and national contexts for decreasing disease burden.

## Supporting information

Choi et al. supplementary material Choi et al. supplementary material

## Data Availability

Data are available at the GBD 2019 data website (https://www.healthdata.org/data-tools-practices/data-sources).
