# Supplementary material for: Differential trends and patterns of sociodemographic disparities in burden of mental disorders, substance use disorder and self-harm across age groups: ecological study in 204 countries using the Global Burden of Disease Study 2019
Source: BJPsych Open. 2024 Apr 19;10(3):e89. doi: 10.1192/bjo.2024.26 (PMC11060086; doi:10.1192/bjo.2024.26)
Supplement: Choi et al. supplementary material [file S2056472424000267sup001.docx]

Supplementary Table 1. International Classifications of Disease (ICD) codes for mental disorders, substance use disorders, and self-harm, 1990-2019

| **Level 2** | **Level 3** | **ICD 10** | **ICD 9** |
| --- | --- | --- | --- |
| Mental disorder | Anxiety disorder | F40-F44.9, F93-F93.2 | 300-300.3, 308-309.9 |
|  | ADHD | F90-F90.9 | 314-314.9 |
|  | Autism spectrum disorder |  |  |
|  | Bipolar disorder | F30-F31.9, F34.0 | 296-296.16, 296.4-296.81 |
|  | Conduct disorder | F91-F92.9 | 312-312.9 |
|  | Depressive disorder | F32-F33.9, F34.1 | 296.2-296.36, 300.4, 311-311.9 |
|  | Eating disorder | F50-F50.9 | 307.1, 307.5-307.59 |
|  | IDID | F70-F79.9, Z81.0 | 317-319.9, V18.4 |
|  | Schizophrenia | F20-F20.9, F25-F25.9 | 295-295.35, 295.5-295.8 |
|  | Other mental disorder | F04-F06.1, F06.3-F07.0, F08-F09.9, F21-F24, F26-F29.9, F34, F34.8-F34.9, F38-F39, F45-F49, F51-F52.9, F55-F55.8, F56-F69.0, F80-F89.0, F93.3-F99.0, G47-G47.29, G47.4-G47.9, R40-R40.4, R45-R46.89, R55-R55.0, Z03.2, Z04.6-Z04.72, Z13.4, Z64, Z81, Z81.8, Z86.5-Z86.59 | 293-294, 295.4-295.45, 295.80-295.95, 296.82-298.9, 300.5-302.9, 306-307.0, 307.2-307.49, 307.6-307.7, 307.9, 310-310.1, 313-313.9, 316-316.9, 327-327.19, 327.3-327.8, 347-347.9, 780-780.2, 780.93, 780.97, 797-797.9, 799.2-799.29, V11.0-V11.2, V11.4-V12.0, V17-V17.0, V40-V41.9, V79-V79.9 |
| Substance use disorder | Alcohol use disorder | E24.4, F10-F10.99, G31.2, G62.1, R78.0, X45-X45.9, X65-X65.9, Y15-Y15.9, Z81.1 | 291-291.9, 303-303.93, 305-305.03, 790.3, E860-E860.19, V11.3 |
|  | Drug use disorder | F11-F19.99, P96.1, R78.1-R78.9, Z81.2-Z81.4 | 292-292.9, 304-304.93, 305.1-305.93, E850.0-E850.29, V15.8-V15.83, V15.85-V15.86, nan |
| Self-harm | Self-harm | X60-X64.9, X66-X84.9, Y87.0 | E950-E959 |

Supplementary Table 2. Lists of 204 countries and territories by sociodemographic index

| **SDI level** | **Locations** |
| --- | --- |
| Low SDI countries  (34 countries) | Afghanistan, Benin, Burkina Faso, Burundi, Central African Republic, Chad, Comoros, Côte d'Ivoire, DR Congo, Eritrea, Ethiopia, Guinea, Guinea-Bissau, Haiti, Liberia, Madagascar, Malawi, Mali, Mozambique, Nepal, Niger, Pakistan, Papua New Guinea, Rwanda, Senegal, Sierra Leone, Solomon Island, Somalia, South Sudan, Tanzania, The Gambia, Togo, Uganda, and Yemen |
| Low-middle SDI countries  (42 countries) | Angola, Bangladesh, Belize, Bhutan, Bolivia, Cambodia, Cameroon, Cape Verde, Congo, Djibouti, Dominican Republic, El Salvador, eSwatini, Federated States of Micronesia, Ghana, Guatemala, Honduras, India, Kenya, Kiribati, Kyrgyzstan, Laos, Lesotho, Maldives, Marshall Islands, Mauritania, Mongolia, Morocco, Myanmar, Nicaragua, Nigeria, North Korea, Palestine, São Tomé and PrÍncipe, Sudan, Tajikistan, Timor-Leste, Tuvalu, Vanuatu, Venezuela, Zambia, and Zimbabwe |
| Middle SDI countries  (41 countries) | Albania, Algeria, Armenia, Azerbaijan, Botswana, Brazil, China, Colombia, Costa Rica, Cuba, Ecuador, Egypt, Equatorial Guinea, Fiji, Gabon, Grenada, Guyana, Indonesia, Iran, Iraq, Jamaica, Mexico, Namibia, Nauru, Panama, Paraguay, Peru, Philippines, Saint Lucia, Saint Vincent and the Grenadines, Samoa, South Africa, Suriname, Syria, Thailand, Tokelau, Tonga, Tunisia, Turkmenistan, Uzbekistan, and Vietnam |
| High-middle SDI countries  (47 countries) | American Samoa, Antigua and Barbuda, Argentina, Bahrain, Barbados, Belarus, Chile, Cook Islands, Croatia, Dominica, Georgia, Greece, Greenland, Hungary, Israel, Italy, Jordan, Kazakhstan, Lebanon, Libya, Malaysia, Malta, Mauritius, Moldova, Montenegro, Niue, North Macedonia, Northern Mariana Islands, Oman, Palau, Poland, Portugal, Romania, Russia, Saint Kitts and Nevis, Serbia, Seychelles, Spain, Sri Lanka, The Bahamas, Trinidad and Tobago, Turkey, Ukraine, and Uruguay, Virgin Islands |
| High SDI countries  (40 countries) | Andorra, Australia, Austria, Belgium, Bermuda, Brunei, Canada, Cyprus, Czech Republic, Denmark, Estonia, Finland, France, Germany, Guam, Iceland, Ireland, Japan, Kuwait, Latvia, Lithuania, Luxembourg, Monaco, Netherlands, New Zealand, Norway, Puerto Rico, Qatar, San Marino, Saudi Arabia, Singapore, Slovakia, Slovenia, South Korea, Sweden, Switzerland, Taiwan, UK, United Arab Emirates, and USA |

Supplementary Table 3. Disability Adjusted Life Years (DALY) rate and 95% uncertainty intervals for mental disorders, substance use disorders, and self-harm between 1990 and 2019 among those aged 10-24 years

|  | **Low SDI** | **Low-middle SDI** | **Middle SDI** | **High-middle SDI** | **High SDI** |
| --- | --- | --- | --- | --- | --- |
| **10-24 years** |  |  |  |  |  |
| Mental disorder |  |  |  |  |  |
| 1990 | 1475.9(1044.1-2006.7) | 1425.3(1017-1944.9) | 1429.2(1021.2-1937.1) | 1510(1076-2040.5) | 1916.6(1360.2-2590.5) |
| 2019 | 1480.7(1040.9-2025.8) | 1400.8(995.3-1907.3) | 1439.4(1021.1-1951.6) | 1527.9(1080.8-2071.1) | 2114.1(1508.4-2860.5) |
| Change % | 0.3(-1-1.4) | -1.7(-3.5-0) | 0.7(-0.8-2.3) | 1.2(-0.5-2.8) | 10.3(8.2-12.6) |
| Anxiety disorder |  |  |  |  |  |
| 1990 | 347.2(225.8-508.4) | 330.5(215.3-477.4) | 426.9(280.8-611.4) | 443.2(291.5-636.7) | 517.5(342.3-748.1) |
| 2019 | 362.7(236.1-537.1) | 333.3(218.7-482.9) | 428.9(284.5-614.4) | 443.9(292.6-638.2) | 547.1(359.1-791) |
| Change % | 4.5(2.2-6.6) | 0.8(-1.5-3.2) | 0.5(-1.7-2.9) | 0.2(-2.7-2.9) | 5.7(3.1-8.3) |
| ADHD |  |  |  |  |  |
| 1990 | 16.8(9.1-29.5) | 22.4(12.5-38.5) | 35.2(19.7-61.5) | 37.6(21.2-64.3) | 38(21.3-65.9) |
| 2019 | 16.8(9.2-29.5) | 21(11.6-37.2) | 32(17.9-55.8) | 37.4(21.1-64) | 41.7(23.6-73.1) |
| Change % | -0.1(-3.3-3.2) | -5.9(-10--1.6) | -8.9(-12.3--4.8) | -0.4(-4.6-5.1) | 9.6(5-14.6) |
| Autism spectrum disorder |  |  |  |  |  |
| 1990 | 58.1(38-84.1) | 53.2(34.9-77.6) | 54.4(35.5-79.8) | 68.9(44.9-99.4) | 91.8(60-131.5) |
| 2019 | 58.8(38.5-85.8) | 53.5(35.2-78.2) | 55.4(36.2-80.9) | 68.9(45-99.6) | 98.9(64.6-140.7) |
| Change % | 1.3(-1.3-3.9) | 0.6(-2.2-3.5) | 1.9(-0.2-4) | 0(-2.4-2.3) | 7.8(5.6-9.9) |
| Bipolar disorder |  |  |  |  |  |
| 1990 | 99.1(51.3-163.3) | 84.6(45.6-136.9) | 87.5(47.5-138) | 102.8(55.3-166.1) | 173.2(99.6-268.7) |
| 2019 | 103(53.6-172.6) | 91.5(49.6-149.8) | 100.6(54.9-162.1) | 114.5(62.5-184.1) | 176.7(103.5-269.4) |
| Change % | 3.9(0.8-6.9) | 8.2(4.9-11.7) | 15(12.2-17.7) | 11.4(8.3-14.5) | 2(-0.5-4.9) |
| Conduct disorder |  |  |  |  |  |
| 1990 | 242(135.6-384.3) | 215.2(118.3-344.3) | 201(111-320.4) | 203.5(111.8-325.9) | 207.8(115.2-331.9) |
| 2019 | 242.7(134.5-387.6) | 216.1(119-344.5) | 210.8(116.8-338.6) | 203.4(112.8-323.9) | 212.7(118.8-343.8) |
| Change % | 0.3(-1.8-2.3) | 0.4(-3-3.8) | 4.9(2.4-7.4) | -0.1(-2.4-2.7) | 2.3(-0.1-4.8) |
| Depressive disorder |  |  |  |  |  |
| 1990 | 483.6(306.1-722.6) | 444.5(283.6-668.2) | 403(259.5-596.4) | 439.8(287.5-643) | 592.1(390.7-860.6) |
| 2019 | 475.4(298.9-713.4) | 425.8(271.9-632.5) | 386.8(248.4-575) | 426.5(274.7-631) | 725.5(473.6-1063.2) |
| Change % | -1.7(-4.1-0.3) | -4.2(-7.7--0.6) | -4(-7.3--1.1) | -3(-7.7-1) | 22.5(18.1-26.9) |
| Eating disorder |  |  |  |  |  |
| 1990 | 30.3(17.9-47.3) | 33.1(19.4-52.1) | 38.7(22.9-60) | 57.8(34.1-89.1) | 130.2(78.1-201.5) |
| 2019 | 34.3(20.2-53.4) | 44.5(26.3-69.4) | 54.8(32.3-85.6) | 76.3(44.5-119.8) | 149.8(89.8-230) |
| Change % | 13.1(7.9-18) | 34.3(28.2-40.1) | 41.4(36.5-46.5) | 31.9(26.2-37.7) | 15(12.1-18.4) |
| IDID |  |  |  |  |  |
| 1990 | 116.2(63-187.7) | 147(81.4-234.4) | 70.9(37-116.8) | 40.5(18.6-69.7) | 25.7(9.8-45.1) |
| 2019 | 102.5(53.8-169.9) | 116.2(63.6-188.8) | 59.5(30.9-99.9) | 34.5(15.7-59.5) | 20.3(6.7-36.6) |
| Change % | -11.8(-17.3--8.2) | -21(-25--17.9) | -16.1(-21.3--12.3) | -14.9(-20.1--11.1) | -21.2(-30.9--16.8) |
| Schizophrenia |  |  |  |  |  |
| 1990 | 49.9(31.9-75.8) | 62(40.1-91.4) | 77.5(51-111.5) | 78.6(52.2-111.1) | 84.5(55.1-123.5) |
| 2019 | 50.7(32-77.9) | 64.2(41.6-96.3) | 75.5(48.8-111.3) | 83.6(55.7-118.7) | 84.8(55.7-124.2) |
| Change % | 1.6(-3.9-7.7) | 3.6(-2-9.2) | -2.6(-7.6-1.8) | 6.3(0.5-11.8) | 0.3(-3.5-4.5) |
| Other mental disorders |  |  |  |  |  |
| 1990 | 32.7(16.9-54.6) | 32.7(16.9-54) | 34.1(17.7-56.4) | 37.2(19.5-61.6) | 55.7(30.3-89.1) |
| 2019 | 33.8(17.8-55.9) | 34.7(18-57.5) | 35.1(18.2-58.2) | 38.9(20.3-64.1) | 56.6(30.8-91.1) |
| Change % | 3.3(-0.9-8.4) | 6.1(1.5-10.6) | 2.9(-0.5-6.6) | 4.5(0.1-8.9) | 1.5(-1.8-5) |
| Substance use disorder |  |  |  |  |  |
| 1990 | 128.7(87.7-176.9) | 220.9(163.6-290.2) | 284.4(209.6-366.2) | 385.3(286.9-497.6) | 573.1(408.5-753.1) |
| 2019 | 130.9(90.5-179.1) | 181.8(130.7-240.5) | 224.8(158.8-300.8) | 332.6(235.5-443.8) | 1035.4(781.5-1320.9) |
| Change % | 1.7(-2.1-5.8) | -17.7(-22.2--13.2) | -21(-25.8--17.1) | -13.7(-18.4--9.3) | 80.7(60.5-105.5) |
| Alcohol use disorder |  |  |  |  |  |
| 1990 | 64.3(39.5-100.1) | 93.5(61.9-138.5) | 80.1(52.4-120.3) | 141.4(97.3-204.5) | 204.3(126.2-313.7) |
| 2019 | 62.3(38.3-97) | 71.7(47-106) | 68.5(43.5-105.2) | 109.3(70.9-164.7) | 174(109.2-269.6) |
| Change % | -3.2(-8.4-1.8) | -23.3(-27.6--18.7) | -14.5(-18.7--11.2) | -22.7(-28.1--17.9) | -14.9(-18.3--11.4) |
| Drug use disorder |  |  |  |  |  |
| 1990 | 64.3(43.4-91.9) | 127.3(93.6-171.6) | 204.3(150-269.1) | 243.9(178.6-323.7) | 368.7(266.7-486.1) |
| 2019 | 68.6(47.7-96.9) | 110(77.9-152.7) | 156.3(108.7-214.6) | 223.3(154.5-308.8) | 861.4(656.8-1106.6) |
| Change % | 6.6(1.2-13.1) | -13.6(-19.9--8.1) | -23.5(-29.4--18.2) | -8.4(-14.4--3) | 133.6(106.4-164.9) |
| Self-harm |  |  |  |  |  |
| 1990 | 454.6(378.4-540.8) | 1196.5(1022.5-1344) | 692.2(580.1-770.6) | 653.9(580.1-710.6) | 591.5(579.6-619.3) |
| 2019 | 315.2(264.6-389.2) | 679.7(596.5-773.1) | 360.6(324.4-403.7) | 396.5(363.2-433.2) | 471.1(456.9-488.8) |
| Change % | -30.7(-42.1--16.4) | -43.2(-51.4--33.4) | -47.9(-54.8--38.4) | -39.4(-46--30.5) | -20.4(-24.9--16.5) |

ADHD: Attention-deficit/hyperactivity disorder

Supplementary Table 4. Disability Adjusted Life Years (DALY) rate and 95% uncertainty intervals for mental disorders, substance use disorders, and self-harm between 1990 and 2019 among those aged 25-49 years

|  | **Low SDI** | **Low-middle SDI** | **Middle SDI** | **Middle-high SDI** | **High SDI** |
| --- | --- | --- | --- | --- | --- |
| **25-49 years** |  |  |  |  |  |
| Mental disorder |  |  |  |  |  |
| 1990 | 2251.6(1633-2951.3) | 2228.3(1627.2-2899.7) | 1946.1(1437.2-2530) | 2010(1481.1-2615.5) | 2443(1796.4-3174.4) |
| 2019 | 2212.6(1607.8-2909.3) | 2146.9(1570.4-2786.6) | 1947(1444-2532.9) | 1950.7(1438.4-2540.3) | 2514.9(1845.9-3268.2) |
| Change % | -1.7(-2.9--0.5) | -3.7(-5.6--1.9) | 0.1(-1.8-1.7) | -3(-4.5--1.4) | 2.9(1.3-4.5) |
| Anxiety disorder |  |  |  |  |  |
| 1990 | 432.2(275.5-621.9) | 424.2(275.2-606.5) | 430(279.8-612.1) | 428.7(280.9-607.6) | 574.3(374.8-808.7) |
| 2019 | 444.7(284.8-637.3) | 441.1(288.9-626.3) | 448.7(295.6-632.1) | 425.4(278.4-599.5) | 588(379.7-834.9) |
| Change % | 2.9(0.9-5.2) | 4(1.6-6.5) | 4.3(0.1-8.1) | -0.8(-4.5-3.2) | 2.4(-0.4-5.2) |
| ADHD |  |  |  |  |  |
| 1990 | 5.5(2.8-9.5) | 7.8(4.1-13.6) | 13.6(7.4-23.5) | 13.9(7.4-24) | 12(6.4-20.5) |
| 2019 | 5.6(2.9-9.7) | 7.5(3.9-13.1) | 11.5(6.1-19.9) | 13.2(7-22.5) | 12.3(6.5-21.2) |
| Change % | 2.3(-1.7-6.5) | -3.9(-7.9-0.6) | -15.4(-19.6--11.3) | -5.3(-10.8-0.5) | 2.3(-1.9-6.6) |
| Autism spectrum disorder |  |  |  |  |  |
| 1990 | 48.6(31.9-70.6) | 45.1(29.5-65) | 47(30.7-68.4) | 60(39-86.6) | 79.7(52.5-113.6) |
| 2019 | 49.1(32.2-71) | 45.1(29.5-65.7) | 46.4(30.3-67) | 59.2(38.7-85.6) | 82.8(54.6-118.9) |
| Change % | 1(-1.3-3.4) | -0.2(-2.2-1.9) | -1.2(-3-0.5) | -1.2(-3.2-0.9) | 3.9(2-5.8) |
| Bipolar disorder |  |  |  |  |  |
| 1990 | 159.4(95.3-250.8) | 132.8(80-210.2) | 128.6(77-203.5) | 151.2(91.1-237.9) | 210(129.5-323.4) |
| 2019 | 164.4(97.4-257.6) | 140.2(83.9-219.3) | 139.7(84-220.3) | 147.1(89.1-231.4) | 206.9(128.6-318.9) |
| Change % | 3.1(1-5.2) | 5.6(3.3-7.7) | 8.7(6.1-11.6) | -2.7(-5.3-0.1) | -1.5(-3.6-0.7) |
| Conduct disorder |  |  |  |  |  |
| 1990 | n/a | n/a | n/a | n/a | n/a |
| 2019 | n/a | n/a | n/a | n/a | n/a |
| Change % | n/a | n/a | n/a | n/a | n/a |
| Depressive disorder |  |  |  |  |  |
| 1990 | 1042.3(699.4-1462.9) | 982.7(660.5-1378.1) | 733.2(496.9-1021.9) | 751.2(509.5-1046.8) | 812.4(559.9-1114.7) |
| 2019 | 987.7(657.4-1397.6) | 889(599.3-1241.1) | 694.9(470.2-975.9) | 673.7(454.9-940) | 864.3(584.1-1199.3) |
| Change % | -5.2(-7--3.4) | -9.5(-12.7--6.6) | -5.2(-8.7--2) | -10.3(-13.3--7.5) | 6.4(2.3-10) |
| Eating disorder |  |  |  |  |  |
| 1990 | 32.8(19.5-50.3) | 34.9(20.8-53.3) | 39.5(23.7-59.9) | 59.8(36.6-89.7) | 134.5(82.2-199.4) |
| 2019 | 38.1(22.5-58.2) | 47.8(28.5-72.4) | 54(32.6-81.8) | 70.6(42.7-105.6) | 144.7(89.9-213.1) |
| Change % | 16.3(11.5-21.2) | 37.1(31.2-43.4) | 36.8(32.2-41.6) | 18(13.8-22) | 7.6(5-11) |
| IDID |  |  |  |  |  |
| 1990 | 91.6(49.3-147.8) | 117.8(65.3-190) | 57(29.6-94.2) | 32.6(15.2-55.5) | 22.9(9.4-39.4) |
| 2019 | 81.5(42.3-134.1) | 87.8(48.1-141.2) | 45(23.3-74.5) | 25.8(11.7-44) | 17.1(5.8-30.4) |
| Change % | -11(-16.3--7.2) | -25.5(-29.1--22.7) | -21.1(-26.4--16.9) | -20.9(-27.3--17.4) | -25.4(-35.3--20.6) |
| Schizophrenia |  |  |  |  |  |
| 1990 | 281.4(195.3-371.2) | 326.4(228.8-428.3) | 340.5(243.8-443.3) | 351.3(251.9-453.3) | 403.9(287-525.7) |
| 2019 | 281.5(195-374.4) | 328.9(229.6-432.5) | 345.3(245.4-450.9) | 369.3(266.4-475.1) | 404.3(286.4-526.2) |
| Change % | 0(-2.5-2.5) | 0.8(-1.5-3.1) | 1.4(-0.8-3.6) | 5.1(2.6-8.1) | 0.1(-1.9-2) |
| Other mental disorders |  |  |  |  |  |
| 1990 | 157.9(99.8-241.8) | 156.7(99.4-242.4) | 156.5(98.9-241.6) | 161.3(101.7-248) | 193.3(122.6-291.2) |
| 2019 | 160(101.8-246.3) | 159.5(101.2-246.3) | 161.4(102.2-249.6) | 166.4(105.2-255.7) | 194.5(124-293.1) |
| Change % | 1.3(-0.4-3.2) | 1.8(0-3.5) | 3.1(1.3-5) | 3.1(0.9-5.3) | 0.6(-1-2.3) |
| Substance use disorder |  |  |  |  |  |
| 1990 | 452.8(338.3-593.8) | 665.1(516-839.3) | 666.9(518.7-836.6) | 1060(865.9-1294.4) | 1012.3(791.9-1272.2) |
| 2019 | 427.8(317.4-557.3) | 570(444.8-719.9) | 541.3(411.7-686.7) | 890.1(712.2-1101.3) | 1931.4(1608.6-2270.3) |
| Change % | -5.5(-9.6--0.1) | -14.3(-18.5--9.3) | -18.8(-22.6--15.4) | -16(-19.6--12.2) | 90.8(74.2-110.2) |
| Alcohol use disorder |  |  |  |  |  |
| 1990 | 308.4(223.5-421) | 427.7(324.2-561.1) | 309.5(232.7-414.8) | 666.8(545.4-826) | 518.9(390.8-682.3) |
| 2019 | 271.6(195.9-372.4) | 359.5(274.8-471.3) | 272.2(201.8-366.1) | 514.5(416.5-649.8) | 449.8(341.7-591.8) |
| Change % | -12(-17.5--5.7) | -16(-21.1--9.2) | -12.1(-17.2--7.2) | -22.8(-27.3--18.9) | -13.3(-16.1--10) |
| Drug use disorder |  |  |  |  |  |
| 1990 | 144.4(102.1-194) | 237.3(177.2-306.2) | 357.4(272.2-453.7) | 393.2(301.8-502.3) | 493.3(386.9-615.2) |
| 2019 | 156.3(114.4-206.9) | 210.5(155.4-275.9) | 269.1(198.6-352.7) | 375.6(284.9-483.5) | 1481.6(1246.8-1738.4) |
| Change % | 8.2(3.4-13.8) | -11.3(-16.4--6.6) | -24.7(-29.6--20.4) | -4.5(-9.1-0.7) | 200.3(172.7-234.7) |
| Self-harm |  |  |  |  |  |
| 1990 | 682.8(590.7-810.8) | 1308.1(1098.2-1458.8) | 957(786.3-1073.5) | 1102.8(1006.6-1176.3) | 1003.7(986.6-1059.3) |
| 2019 | 539.3(449.3-653) | 910.3(774.5-1039.1) | 458(402.6-516.8) | 634.1(582.9-705.8) | 802.1(767.9-834) |
| Change % | -21(-32.8--6.6) | -30.4(-39.1--20) | -52.1(-59--42.2) | -42.5(-48.6--35) | -20.1(-25.8--16.4) |

ADHD: Attention-deficit/hyperactivity disorder

n/a: showing specific mental disorders for which the DALY rate was not estimated

Supplementary Table 5. Disability Adjusted Life Years (DALY) rate and 95% uncertainty intervals for mental disorders, substance use disorders, and self-harm between 1990 and 2019 among those aged 50-69 years

|  | **Low SDI** | **Low-middle SDI** | **Middle SDI** | **Middle-high SDI** | **High SDI** |
| --- | --- | --- | --- | --- | --- |
| **50-69 years** |  |  |  |  |  |
| Mental disorder |  |  |  |  |  |
| 1990 | 2341(1714.2-3119.6) | 2258.9(1679.2-2991.4) | 1906.6(1421.5-2507.5) | 2023.6(1497.1-2652.7) | 2050.7(1524.4-2677.6) |
| 2019 | 2322.6(1704.7-3103) | 2251.1(1659.6-2983.3) | 1966.9(1459.5-2590.6) | 1985.9(1472.7-2595.6) | 2054.8(1528.4-2684.1) |
| Change % | -0.8(-2.1-0.5) | -0.3(-1.7-1.2) | 3.2(1.8-4.5) | -1.9(-3.2--0.6) | 0.2(-1.2-1.7) |
| Anxiety disorder |  |  |  |  |  |
| 1990 | 374.4(251.9-534.4) | 401.7(273.1-562) | 424(290.1-589) | 428.5(291.6-602.3) | 492.2(334-697.6) |
| 2019 | 386.5(261.4-550) | 420(290.4-586.8) | 426.9(292.2-593.9) | 422(288.9-591.5) | 511.2(346.6-725.7) |
| Change % | 3.2(0.9-5.9) | 4.6(2.3-6.9) | 0.7(-1.3-2.7) | -1.5(-3.6-0.7) | 3.9(1.4-6.9) |
| ADHD |  |  |  |  |  |
| 1990 | 1.4(0.7-2.6) | 2.1(1-3.8) | 4(2-7.1) | 3.7(1.8-6.5) | 3(1.4-5.4) |
| 2019 | 1.4(0.7-2.7) | 2.2(1-4) | 3.6(1.8-6.5) | 3.8(1.8-6.7) | 3.2(1.6-5.8) |
| Change % | 1.8(-6.1-11.2) | 5.9(-1.2-14) | -9.9(-15.6--3.8) | 3.1(-4-10.6) | 7.4(1.6-13) |
| Autism spectrum disorder |  |  |  |  |  |
| 1990 | 39.7(26.2-57.8) | 36.4(24.2-52.6) | 38.1(25-55.5) | 48.7(32.5-69.8) | 65(43.2-92.4) |
| 2019 | 39.4(26.1-57.3) | 36.2(24-52.4) | 37.6(25-54.6) | 48.5(32.2-69.8) | 69.2(46.1-97.7) |
| Change % | -0.8(-3.4-1.7) | -0.4(-3-2.4) | -1.1(-3.5-1.2) | -0.3(-2.6-2) | 6.5(4.3-8.5) |
| Bipolar disorder |  |  |  |  |  |
| 1990 | 152(90.9-240.5) | 127(76.9-198.3) | 114.2(69.3-178.1) | 149.1(90.8-232.4) | 196.5(121.8-298.9) |
| 2019 | 153.2(91.4-244.9) | 130.8(78.9-206.4) | 117.8(71-185.3) | 133.5(80.8-209.1) | 190.7(118.5-290.7) |
| Change % | 0.8(-1.6-3.1) | 3(0.6-5.4) | 3.1(1.4-5) | -10.5(-12.2--8.7) | -2.9(-4.9--0.9) |
| Conduct disorder |  |  |  |  |  |
| 1990 | n/a | n/a | n/a | n/a | n/a |
| 2019 | n/a | n/a | n/a | n/a | n/a |
| Change % | n/a | n/a | n/a | n/a | n/a |
| Depressive disorder |  |  |  |  |  |
| 1990 | 1299.5(872.7-1855.8) | 1168.4(802.9-1674.3) | 835.7(573.4-1171) | 907.7(621.4-1268.2) | 764(532.5-1068.3) |
| 2019 | 1264.7(850.5-1798.8) | 1153.9(792.8-1630.5) | 896.8(617-1252.6) | 889(607.1-1241.9) | 746.1(513-1051.7) |
| Change % | -2.7(-4.7--0.6) | -1.2(-3.5-1.3) | 7.3(5-9.6) | -2.1(-4.4-0.6) | -2.3(-5.9-1.1) |
| Eating disorder |  |  |  |  |  |
| 1990 | n/a | n/a | n/a | n/a | n/a |
| 2019 | n/a | n/a | n/a | n/a | n/a |
| Change % | n/a | n/a | n/a | n/a | n/a |
| IDID |  |  |  |  |  |
| 1990 | 61.6(33.2-100.1) | 80.1(44.3-129.9) | 39.8(20.7-65.3) | 23.8(11.4-39.7) | 18.7(8.5-31.4) |
| 2019 | 59.4(31.2-96.7) | 60(33.2-97.2) | 28.6(14.4-47.5) | 17.3(7.5-29.6) | 13.5(5.4-23.2) |
| Change % | -3.5(-8.8-0.5) | -25.1(-28.6--21.8) | -28.1(-35.3--23.3) | -27.6(-36.1--22.9) | -28.1(-38.1--22.6) |
| Schizophrenia |  |  |  |  |  |
| 1990 | 225.8(162.5-289.1) | 257.3(185.1-329.7) | 262.5(192.2-336.2) | 275.3(199.2-348.8) | 314.1(225.4-401.1) |
| 2019 | 231.1(164.6-298.3) | 262.6(187.7-337.5) | 267.7(193-341.8) | 283.4(206.3-358) | 321.6(231-411.7) |
| Change % | 2.4(-0.7-5.6) | 2.1(-0.9-5.2) | 2(-0.3-4.5) | 2.9(0.6-5.4) | 2.4(0.5-4.5) |
| Other mental disorders |  |  |  |  |  |
| 1990 | 186.5(120.6-279.2) | 186(119.3-280.3) | 188.3(120.2-284.4) | 186.8(119.3-281.1) | 197.2(127.7-291.9) |
| 2019 | 186.7(120.8-279.9) | 185.4(118.6-279) | 187.7(119.6-282.9) | 188.4(120.5-284) | 199.3(128.9-294.5) |
| Change % | 0.1(-1.8-2) | -0.3(-2-1.5) | -0.4(-1.9-1.1) | 0.9(-0.7-2.3) | 1(-0.4-2.5) |
| Substance use disorder |  |  |  |  |  |
| 1990 | 389.3(307.3-480.6) | 494.4(397.8-599.1) | 383.8(317.9-465.9) | 791.3(684.3-920.2) | 532.2(449.2-631.5) |
| 2019 | 356.6(280.6-445.4) | 452.6(364.1-555.9) | 335(268.2-417.6) | 592.6(496.8-712.6) | 908.8(788.3-1041.4) |
| Change % | -8.4(-16.8-2.3) | -8.5(-16--0.4) | -12.7(-19.3--7.2) | -25.1(-29.5--20.8) | 70.8(61.4-81.4) |
| Alcohol use disorder |  |  |  |  |  |
| 1990 | 325(251.6-410.6) | 379(295.2-468.3) | 240.5(193.5-302.8) | 639.6(552-749.3) | 417.3(351.7-498.1) |
| 2019 | 286.2(221-367) | 341(265.6-430.9) | 219.8(171.5-280.2) | 463.3(387.6-562) | 448.9(376.9-538.7) |
| Change % | -11.9(-21.4-0.2) | -10(-18.1--0.3) | -8.6(-15.9--2.1) | -27.6(-32.4--23.1) | 7.6(3-11.7) |
| Drug use disorder |  |  |  |  |  |
| 1990 | 64.3(48.6-83.9) | 115.3(92.9-143.6) | 143.4(115.4-177) | 151.7(122.7-187.5) | 114.8(87.5-144.7) |
| 2019 | 70.4(53.5-90.3) | 111.5(87.6-140.3) | 115.2(86.2-151.6) | 129.3(95.6-169.7) | 459.8(388-533.7) |
| Change % | 9.5(2.2-17.8) | -3.3(-11.6-5.1) | -19.7(-29.3--12) | -14.8(-22.8--8) | 300.5(256.5-360.6) |
| Self-harm |  |  |  |  |  |
| 1990 | 761.2(640.5-925.5) | 756.7(627.1-881.1) | 706.2(578.7-801.7) | 906.2(847.9-957.1) | 748.7(732-787.8) |
| 2019 | 581.6(478.2-700) | 532.6(443.2-615.9) | 330.7(287.9-377.5) | 428(388.7-483.9) | 628.8(598-658.4) |
| Change % | -23.6(-34.4--9.2) | -29.6(-38.8--15) | -53.2(-59.3--44.4) | -52.8(-57.2--46.8) | -16(-22.4--11.9) |

ADHD: Attention-deficit/hyperactivity disorder

n/a: showing specific mental disorders for which the DALY rate was not estimated

Supplementary Table 6. Disability Adjusted Life Years (DALY) rate and 95% uncertainty intervals for mental disorders, substance use disorders, and self-harm between 1990 and 2019 among those over 70 years

|  | **Low SDI** | **Low-middle SDI** | **Middle SDI** | **Middle-high SDI** | **High SDI** |
| --- | --- | --- | --- | --- | --- |
| **Over 70 years** |  |  |  |  |  |
| Mental disorder |  |  |  |  |  |
| 1990 | 2063.8(1489.5-2704.7) | 1822.6(1335.6-2357.7) | 1576.1(1171.1-2018.6) | 1762.8(1301.4-2269.2) | 1636.7(1227.8-2093.4) |
| 2019 | 2025.4(1465.9-2636.6) | 1859.9(1369.4-2406) | 1642.7(1214.8-2109.7) | 1711.8(1266.8-2205.2) | 1526.1(1145.1-1963.4) |
| Change % | -1.9(-3.4--0.3) | 2.0(0.4-3.7) | 4.2(2.6-6) | -2.9(-4.6--1.2) | -6.8(-8.7--4.8) |
| Anxiety disorder |  |  |  |  |  |
| 1990 | 301(197.7-447.8) | 338.1(227.3-487.4) | 388.2(262-551.5) | 399.8(268.6-575.1) | 410.7(276.1-598.4) |
| 2019 | 307(202-452.6) | 344.3(234.2-494.3) | 377.5(256.5-533.1) | 387(261.2-553.8) | 386.1(259.1-562.3) |
| Change % | 2(-1.1-5.1) | 1.8(-0.8-4.7) | -2.8(-5.1--0.2) | -3.2(-5.7--0.3) | -6(-9--2.2) |
| ADHD |  |  |  |  |  |
| 1990 | 0.1(0-0.4) | 0.2(0.1-0.5) | 0.4(0.1-0.9) | 0.3(0.1-0.8) | 0.2(0.1-0.5) |
| 2019 | 0.1(0-0.3) | 0.2(0.1-0.5) | 0.3(0.1-0.8) | 0.3(0.1-0.8) | 0.2(0.1-0.5) |
| Change % | -11.5(-23.5-1.3) | -7.2(-22.7-11.2) | -15.9(-32-2.5) | -2.4(-21.8-17.5) | -0.4(-13.3-15) |
| Autism spectrum disorder |  |  |  |  |  |
| 1990 | 32.7(21.8-46.9) | 29.3(19.6-42) | 29.3(19.7-42.5) | 37.2(25.2-53.1) | 46.9(31.7-66.3) |
| 2019 | 31.3(21.2-45.1) | 28.5(19.2-41.3) | 29.7(19.8-42.9) | 38.5(25.9-55.4) | 54.3(37-77.2) |
| Change % | -4.1(-7.6--0.6) | -2.6(-6.1-1.1) | 1.3(-1.5-4.2) | 3.5(0.8-6) | 15.9(13.3-18.4) |
| Bipolar disorder |  |  |  |  |  |
| 1990 | 90.1(52.2-142.8) | 76.7(46-117.9) | 71.5(42.8-109.1) | 97.1(58.6-148.7) | 128.9(78.9-191.9) |
| 2019 | 86.2(50.9-134.4) | 74(44.4-114.2) | 70.1(42.2-106.4) | 89.4(53.6-136.2) | 121.4(74.6-184) |
| Change % | -4.4(-7.9--0.6) | -3.6(-6.9-0.3) | -2(-5-1.1) | -7.9(-10.5--5.1) | -5.9(-8.4--2.8) |
| Conduct disorder |  |  |  |  |  |
| 1990 | n/a | n/a | n/a | n/a | n/a |
| 2019 | n/a | n/a | n/a | n/a | n/a |
| Change % | n/a | n/a | n/a | n/a | n/a |
| Depressive disorder |  |  |  |  |  |
| 1990 | 1340.3(907.2-1871.6) | 1050.6(717.4-1439.9) | 770.5(527.2-1051.6) | 914.4(621-1255.8) | 725.7(503-973) |
| 2019 | 1288.3(875.8-1792.7) | 1082(742-1471.9) | 849.7(583.1-1150.8) | 882.2(600.4-1191.8) | 644.8(448.4-876.4) |
| Change % | -3.9(-5.9--1.9) | 3(0.3-5.7) | 10.3(7.8-12.7) | -3.5(-6.2--0.8) | -11.2(-14.8--7.5) |
| Eating disorder |  |  |  |  |  |
| 1990 | n/a | n/a | n/a | n/a | n/a |
| 2019 | n/a | n/a | n/a | n/a | n/a |
| Change % | n/a | n/a | n/a | n/a | n/a |
| IDID |  |  |  |  |  |
| 1990 | 31.1(16.6-50.9) | 41.8(22.4-67.5) | 22.5(11.8-36.9) | 16.3(8.4-26) | 13.6(7.1-21.9) |
| 2019 | 38.5(20.6-62.2) | 38.6(22-60.4) | 17.4(9-28.3) | 11.4(5.6-19) | 8.1(3.5-14) |
| Change % | 23.9(17.4-32.4) | -7.8(-13.5-1.9) | -22.7(-29--15.6) | -30.3(-38--25.5) | -40.7(-51.3--33.6) |
| Schizophrenia |  |  |  |  |  |
| 1990 | 90.2(64.1-117.8) | 108.2(78.4-139.5) | 115.4(83.5-149.1) | 122(88.5-155.1) | 132.1(95.2-171.6) |
| 2019 | 95.6(68.2-125.7) | 115.1(83-148.1) | 118.4(85.4-153.5) | 123.8(90-158.4) | 128.4(92.9-166.2) |
| Change % | 6(0.5-12) | 6.4(1.7-11.4) | 2.6(-1.4-6.8) | 1.4(-1.8-4.9) | -2.8(-5.7-0.5) |
| Other mental disorders |  |  |  |  |  |
| 1990 | 178.2(120.1-257.3) | 177.6(119.9-254.7) | 178.3(120.2-257.2) | 175.6(118-253.7) | 178.5(120.2-257.7) |
| 2019 | 178.3(120.1-255.2) | 177.2(119.4-253.7) | 179.6(120.3-259.4) | 179.2(121.6-258.4) | 182.7(122.9-263.2) |
| Change % | 0(-2.1-2.3) | -0.3(-2.2-1.7) | 0.7(-1-2.5) | 2.1(0.3-3.8) | 2.4(0.7-4.1) |
| Substance use disorder |  |  |  |  |  |
| 1990 | 241.6(195.4-292.6) | 277.3(226.5-337.1) | 244.5(203-294.8) | 292.6(238.5-359) | 219.4(179.9-266.3) |
| 2019 | 250.3(204-308.7) | 286.2(230.7-355) | 238.8(190.9-298.2) | 275.9(220.9-345.3) | 290.2(243.5-348.3) |
| Change % | 3.6(-4-11.6) | 3.2(-3.8-10.8) | -2.3(-9.7-4.1) | -5.7(-10.1--1.6) | 32.3(26.9-38.3) |
| Alcohol use disorder |  |  |  |  |  |
| 1990 | 195.6(155.1-245) | 195.3(154.2-247.2) | 120.9(96.5-153.7) | 203.8(159-262.4) | 155.5(126.8-191.2) |
| 2019 | 200.5(157.8-255.4) | 206.8(160.3-265.1) | 130(100.8-168.6) | 178.3(138.3-229.7) | 179.3(146-221.3) |
| Change % | 2.5(-5.6-11.6) | 5.9(-2.6-15.2) | 7.5(-0.4-14) | -12.5(-16.3--9.6) | 15.3(11-19.8) |
| Drug use disorder |  |  |  |  |  |
| 1990 | 46(36.9-57.3) | 82(67.2-100.6) | 123.6(98.8-153.3) | 88.8(69.4-110.6) | 63.9(46.8-82.9) |
| 2019 | 49.9(40.8-60) | 79.4(63.2-98.4) | 108.9(80.4-142.7) | 97.6(71.4-127.7) | 110.9(87.9-137) |
| Change % | 8.3(-2.5-20.8) | -3.1(-12.2-4.8) | -11.9(-21.7--3.5) | 10(0.8-17.8) | 73.7(57-93.7) |
| Self-harm |  |  |  |  |  |
| 1990 | 646.9(545.6-801.4) | 505.2(435.9-592.1) | 731(635.4-816.5) | 682(629.1-730.8) | 511.9(485.3-531.1) |
| 2019 | 515.8(437.7-617.8) | 387.4(331.1-442.8) | 393(333.3-451.6) | 330.7(298.3-373.5) | 353.5(322.8-377.3) |
| Change % | -20.3(-28.2--9) | -23.3(-31.8--9.2) | -46.2(-53--36.5) | -51.5(-55.9--44.8) | -30.9(-35.2--26.9) |

ADHD: Attention-deficit/hyperactivity disorder

n/a: showing specific mental disorders for which the DALY rate was not estimated

Supplementary Table 7. Concentration index by disease categories from 1990 to 2019 among those aged 10-24 years

| **Year** | **Mental disorder** | **Anxiety disorder** | **Depressive disorder** | **Schizophrenia** | **Substance use disorder** | **Alcohol use disorder** | **Drug use disorder** | **Self-harm** |
| --- | --- | --- | --- | --- | --- | --- | --- | --- |
| 1990 | 0.04 | 0.07 | 0.02 | 0.07 | 0.27 | 0.26 | 0.28 | 0.1 |
| 1991 | 0.04 | 0.07 | 0.02 | 0.07 | 0.28 | 0.26 | 0.29 | 0.1 |
| 1992 | 0.04 | 0.07 | 0.02 | 0.07 | 0.28 | 0.26 | 0.3 | 0.09 |
| 1993 | 0.04 | 0.07 | 0.02 | 0.07 | 0.28 | 0.26 | 0.3 | 0.09 |
| 1994 | 0.04 | 0.07 | 0.02 | 0.07 | 0.29 | 0.26 | 0.31 | 0.1 |
| 1995 | 0.04 | 0.07 | 0.02 | 0.07 | 0.29 | 0.25 | 0.31 | 0.09 |
| 1996 | 0.05 | 0.07 | 0.02 | 0.07 | 0.29 | 0.25 | 0.31 | 0.09 |
| 1997 | 0.05 | 0.07 | 0.02 | 0.07 | 0.29 | 0.24 | 0.31 | 0.08 |
| 1998 | 0.05 | 0.07 | 0.02 | 0.07 | 0.28 | 0.24 | 0.31 | 0.08 |
| 1999 | 0.05 | 0.07 | 0.02 | 0.07 | 0.28 | 0.24 | 0.32 | 0.08 |
| 2000 | 0.05 | 0.07 | 0.02 | 0.07 | 0.29 | 0.23 | 0.32 | 0.08 |
| 2001 | 0.05 | 0.07 | 0.02 | 0.07 | 0.28 | 0.23 | 0.32 | 0.07 |
| 2002 | 0.05 | 0.07 | 0.02 | 0.07 | 0.28 | 0.23 | 0.32 | 0.07 |
| 2003 | 0.05 | 0.07 | 0.02 | 0.07 | 0.28 | 0.23 | 0.32 | 0.06 |
| 2004 | 0.05 | 0.07 | 0.02 | 0.07 | 0.28 | 0.23 | 0.32 | 0.06 |
| 2005 | 0.05 | 0.07 | 0.02 | 0.07 | 0.28 | 0.23 | 0.32 | 0.05 |
| 2006 | 0.05 | 0.07 | 0.02 | 0.07 | 0.28 | 0.22 | 0.32 | 0.05 |
| 2007 | 0.05 | 0.08 | 0.02 | 0.07 | 0.28 | 0.22 | 0.32 | 0.05 |
| 2008 | 0.05 | 0.08 | 0.02 | 0.07 | 0.28 | 0.22 | 0.32 | 0.05 |
| 2009 | 0.05 | 0.08 | 0.02 | 0.07 | 0.28 | 0.22 | 0.31 | 0.05 |
| 2010 | 0.05 | 0.08 | 0.03 | 0.07 | 0.28 | 0.21 | 0.31 | 0.04 |
| 2011 | 0.05 | 0.08 | 0.03 | 0.07 | 0.27 | 0.21 | 0.31 | 0.04 |
| 2012 | 0.05 | 0.08 | 0.03 | 0.07 | 0.27 | 0.21 | 0.31 | 0.03 |
| 2013 | 0.05 | 0.08 | 0.03 | 0.07 | 0.27 | 0.21 | 0.3 | 0.03 |
| 2014 | 0.05 | 0.08 | 0.03 | 0.07 | 0.27 | 0.21 | 0.3 | 0.02 |
| 2015 | 0.05 | 0.08 | 0.03 | 0.07 | 0.26 | 0.2 | 0.3 | 0.02 |
| 2016 | 0.05 | 0.08 | 0.03 | 0.06 | 0.26 | 0.2 | 0.29 | 0.02 |
| 2017 | 0.05 | 0.07 | 0.03 | 0.06 | 0.26 | 0.16 | 0.29 | 0.01 |
| 2018 | 0.05 | 0.07 | 0.03 | 0.06 | 0.26 | 0.16 | 0.29 | 0.01 |
| 2019 | 0.05 | 0.07 | 0.03 | 0.06 | 0.26 | 0.16 | 0.29 | 0.01 |

Supplementary Table 8. Concentration index by disease categories from 1990 to 2019 among those aged 25-49 years

| **Year** | **Mental disorder** | **Anxiety disorder** | **Depressive disorder** | **Schizophrenia** | **Substance use disorder** | **Alcohol use disorder** | **Drug use disorder** | **Self-harm** |
| --- | --- | --- | --- | --- | --- | --- | --- | --- |
| 1990 | 0.01 | 0.04 | -0.05 | 0.06 | 0.16 | 0.16 | 0.18 | 0.13 |
| 1991 | 0.01 | 0.04 | -0.05 | 0.06 | 0.17 | 0.16 | 0.19 | 0.13 |
| 1992 | 0.01 | 0.04 | -0.05 | 0.06 | 0.18 | 0.16 | 0.2 | 0.13 |
| 1993 | 0.01 | 0.04 | -0.05 | 0.06 | 0.18 | 0.17 | 0.21 | 0.13 |
| 1994 | 0.01 | 0.04 | -0.05 | 0.06 | 0.19 | 0.17 | 0.21 | 0.13 |
| 1995 | 0.01 | 0.04 | -0.05 | 0.06 | 0.19 | 0.17 | 0.22 | 0.13 |
| 1996 | 0.01 | 0.04 | -0.05 | 0.06 | 0.19 | 0.17 | 0.22 | 0.12 |
| 1997 | 0.01 | 0.04 | -0.05 | 0.06 | 0.19 | 0.17 | 0.23 | 0.11 |
| 1998 | 0.01 | 0.04 | -0.05 | 0.06 | 0.19 | 0.16 | 0.23 | 0.11 |
| 1999 | 0.02 | 0.04 | -0.05 | 0.06 | 0.19 | 0.16 | 0.23 | 0.1 |
| 2000 | 0.02 | 0.04 | -0.05 | 0.06 | 0.19 | 0.16 | 0.24 | 0.1 |
| 2001 | 0.02 | 0.04 | -0.05 | 0.06 | 0.19 | 0.16 | 0.24 | 0.09 |
| 2002 | 0.02 | 0.04 | -0.05 | 0.06 | 0.19 | 0.16 | 0.24 | 0.09 |
| 2003 | 0.02 | 0.04 | -0.05 | 0.06 | 0.19 | 0.16 | 0.24 | 0.09 |
| 2004 | 0.01 | 0.04 | -0.05 | 0.06 | 0.19 | 0.15 | 0.24 | 0.08 |
| 2005 | 0.01 | 0.04 | -0.05 | 0.06 | 0.19 | 0.15 | 0.24 | 0.08 |
| 2006 | 0.01 | 0.04 | -0.05 | 0.06 | 0.19 | 0.15 | 0.24 | 0.08 |
| 2007 | 0.02 | 0.04 | -0.05 | 0.06 | 0.19 | 0.15 | 0.25 | 0.08 |
| 2008 | 0.02 | 0.04 | -0.05 | 0.06 | 0.19 | 0.14 | 0.25 | 0.08 |
| 2009 | 0.02 | 0.04 | -0.05 | 0.06 | 0.18 | 0.14 | 0.25 | 0.07 |
| 2010 | 0.02 | 0.04 | -0.05 | 0.06 | 0.18 | 0.13 | 0.25 | 0.07 |
| 2011 | 0.02 | 0.04 | -0.05 | 0.06 | 0.18 | 0.13 | 0.25 | 0.06 |
| 2012 | 0.02 | 0.04 | -0.05 | 0.06 | 0.18 | 0.13 | 0.25 | 0.06 |
| 2013 | 0.02 | 0.04 | -0.05 | 0.06 | 0.18 | 0.13 | 0.25 | 0.05 |
| 2014 | 0.02 | 0.04 | -0.05 | 0.06 | 0.18 | 0.12 | 0.26 | 0.05 |
| 2015 | 0.02 | 0.04 | -0.05 | 0.06 | 0.18 | 0.12 | 0.26 | 0.05 |
| 2016 | 0.02 | 0.04 | -0.05 | 0.06 | 0.19 | 0.12 | 0.26 | 0.04 |
| 2017 | 0.02 | 0.04 | -0.04 | 0.06 | 0.19 | 0.12 | 0.27 | 0.04 |
| 2018 | 0.02 | 0.04 | -0.04 | 0.06 | 0.19 | 0.12 | 0.27 | 0.04 |
| 2019 | 0.02 | 0.04 | -0.04 | 0.06 | 0.19 | 0.12 | 0.27 | 0.04 |

Supplementary Table 9. Concentration index by disease categories from 1990 to 2019 among those aged 50-69 years

| **Year** | **Mental disorder** | **Anxiety disorder** | **Depressive disorder** | **Schizophrenia** | **Substance use disorder** | **Alcohol use disorder** | **Drug use disorder** | **Self-harm** |
| --- | --- | --- | --- | --- | --- | --- | --- | --- |
| 1990 | -0.01 | 0.05 | -0.08 | 0.07 | 0.11 | 0.11 | 0.09 | 0 |
| 1991 | -0.01 | 0.05 | -0.08 | 0.07 | 0.11 | 0.11 | 0.1 | 0 |
| 1992 | -0.01 | 0.05 | -0.08 | 0.07 | 0.12 | 0.12 | 0.1 | 0 |
| 1993 | -0.01 | 0.05 | -0.08 | 0.07 | 0.12 | 0.12 | 0.11 | 0 |
| 1994 | -0.02 | 0.05 | -0.08 | 0.07 | 0.13 | 0.13 | 0.12 | 0.01 |
| 1995 | -0.02 | 0.05 | -0.08 | 0.07 | 0.13 | 0.13 | 0.12 | 0 |
| 1996 | -0.02 | 0.05 | -0.08 | 0.07 | 0.13 | 0.13 | 0.13 | -0.01 |
| 1997 | -0.02 | 0.05 | -0.08 | 0.07 | 0.13 | 0.13 | 0.13 | -0.01 |
| 1998 | -0.02 | 0.05 | -0.08 | 0.07 | 0.13 | 0.13 | 0.13 | -0.02 |
| 1999 | -0.02 | 0.05 | -0.08 | 0.08 | 0.13 | 0.13 | 0.14 | -0.02 |
| 2000 | -0.02 | 0.05 | -0.08 | 0.08 | 0.14 | 0.14 | 0.15 | -0.02 |
| 2001 | -0.02 | 0.05 | -0.09 | 0.08 | 0.14 | 0.14 | 0.15 | -0.02 |
| 2002 | -0.02 | 0.05 | -0.09 | 0.08 | 0.14 | 0.14 | 0.16 | -0.02 |
| 2003 | -0.02 | 0.05 | -0.09 | 0.08 | 0.14 | 0.14 | 0.16 | -0.03 |
| 2004 | -0.02 | 0.05 | -0.09 | 0.08 | 0.14 | 0.14 | 0.16 | -0.03 |
| 2005 | -0.02 | 0.05 | -0.09 | 0.07 | 0.15 | 0.14 | 0.17 | -0.03 |
| 2006 | -0.02 | 0.05 | -0.09 | 0.07 | 0.15 | 0.14 | 0.17 | -0.03 |
| 2007 | -0.02 | 0.05 | -0.09 | 0.07 | 0.15 | 0.14 | 0.17 | -0.03 |
| 2008 | -0.02 | 0.05 | -0.09 | 0.07 | 0.15 | 0.14 | 0.17 | -0.03 |
| 2009 | -0.02 | 0.05 | -0.09 | 0.07 | 0.14 | 0.14 | 0.17 | -0.03 |
| 2010 | -0.02 | 0.05 | -0.09 | 0.07 | 0.14 | 0.13 | 0.17 | -0.04 |
| 2011 | -0.02 | 0.05 | -0.09 | 0.07 | 0.14 | 0.13 | 0.17 | -0.04 |
| 2012 | -0.02 | 0.05 | -0.09 | 0.07 | 0.14 | 0.13 | 0.17 | -0.04 |
| 2013 | -0.02 | 0.05 | -0.09 | 0.07 | 0.14 | 0.13 | 0.18 | -0.05 |
| 2014 | -0.02 | 0.05 | -0.09 | 0.07 | 0.14 | 0.13 | 0.18 | -0.05 |
| 2015 | -0.02 | 0.05 | -0.09 | 0.07 | 0.14 | 0.13 | 0.18 | -0.05 |
| 2016 | -0.02 | 0.05 | -0.09 | 0.07 | 0.14 | 0.13 | 0.18 | -0.05 |
| 2017 | -0.02 | 0.05 | -0.09 | 0.07 | 0.14 | 0.13 | 0.18 | -0.06 |
| 2018 | -0.02 | 0.05 | -0.09 | 0.07 | 0.14 | 0.12 | 0.18 | -0.06 |
| 2019 | -0.02 | 0.05 | -0.09 | 0.07 | 0.14 | 0.12 | 0.19 | -0.06 |

Supplementary Table 10. Concentration index by disease categories from 1990 to 2019 among those over 70 years

| **Year** | **Mental disorder** | **Anxiety disorder** | **Depressive disorder** | **Schizophrenia** | **Substance use disorder** | **Alcohol use disorder** | **Drug use disorder** | **Self-harm** |
| --- | --- | --- | --- | --- | --- | --- | --- | --- |
| 1990 | -0.04 | 0.06 | -0.11 | 0.1 | 0.03 | 0.03 | 0.03 | -0.08 |
| 1991 | -0.04 | 0.06 | -0.11 | 0.1 | 0.03 | 0.03 | 0.03 | -0.09 |
| 1992 | -0.04 | 0.06 | -0.11 | 0.1 | 0.03 | 0.03 | 0.04 | -0.09 |
| 1993 | -0.04 | 0.06 | -0.11 | 0.1 | 0.03 | 0.03 | 0.04 | -0.09 |
| 1994 | -0.04 | 0.06 | -0.11 | 0.1 | 0.03 | 0.03 | 0.04 | -0.1 |
| 1995 | -0.04 | 0.06 | -0.12 | 0.1 | 0.03 | 0.03 | 0.05 | -0.1 |
| 1996 | -0.04 | 0.06 | -0.12 | 0.1 | 0.03 | 0.03 | 0.05 | -0.11 |
| 1997 | -0.04 | 0.06 | -0.12 | 0.1 | 0.03 | 0.03 | 0.06 | -0.11 |
| 1998 | -0.04 | 0.06 | -0.12 | 0.1 | 0.04 | 0.03 | 0.06 | -0.12 |
| 1999 | -0.04 | 0.06 | -0.12 | 0.1 | 0.04 | 0.03 | 0.06 | -0.12 |
| 2000 | -0.04 | 0.06 | -0.12 | 0.1 | 0.04 | 0.03 | 0.06 | -0.13 |
| 2001 | -0.05 | 0.06 | -0.12 | 0.1 | 0.04 | 0.03 | 0.07 | -0.13 |
| 2002 | -0.05 | 0.06 | -0.13 | 0.1 | 0.04 | 0.03 | 0.07 | -0.13 |
| 2003 | -0.05 | 0.06 | -0.13 | 0.1 | 0.04 | 0.03 | 0.07 | -0.13 |
| 2004 | -0.05 | 0.05 | -0.13 | 0.1 | 0.04 | 0.03 | 0.07 | -0.14 |
| 2005 | -0.05 | 0.05 | -0.13 | 0.1 | 0.04 | 0.03 | 0.08 | -0.14 |
| 2006 | -0.05 | 0.05 | -0.13 | 0.09 | 0.04 | 0.03 | 0.08 | -0.14 |
| 2007 | -0.05 | 0.05 | -0.13 | 0.09 | 0.05 | 0.04 | 0.08 | -0.15 |
| 2008 | -0.05 | 0.05 | -0.13 | 0.09 | 0.05 | 0.04 | 0.08 | -0.15 |
| 2009 | -0.05 | 0.06 | -0.13 | 0.09 | 0.05 | 0.04 | 0.08 | -0.15 |
| 2010 | -0.05 | 0.06 | -0.13 | 0.09 | 0.05 | 0.04 | 0.08 | -0.16 |
| 2011 | -0.05 | 0.05 | -0.13 | 0.09 | 0.05 | 0.04 | 0.08 | -0.16 |
| 2012 | -0.05 | 0.05 | -0.13 | 0.09 | 0.05 | 0.04 | 0.08 | -0.16 |
| 2013 | -0.05 | 0.05 | -0.13 | 0.09 | 0.05 | 0.04 | 0.08 | -0.16 |
| 2014 | -0.05 | 0.05 | -0.13 | 0.09 | 0.06 | 0.05 | 0.08 | -0.16 |
| 2015 | -0.05 | 0.05 | -0.13 | 0.09 | 0.06 | 0.05 | 0.08 | -0.16 |
| 2016 | -0.05 | 0.05 | -0.13 | 0.09 | 0.06 | 0.05 | 0.08 | -0.16 |
| 2017 | -0.05 | 0.05 | -0.13 | 0.09 | 0.05 | 0.05 | 0.08 | -0.16 |
| 2018 | -0.05 | 0.05 | -0.13 | 0.09 | 0.05 | 0.04 | 0.08 | -0.16 |
| 2019 | -0.05 | 0.05 | -0.13 | 0.09 | 0.05 | 0.04 | 0.08 | -0.16 |
